# Global health enters into its teenage years

**DOI:** 10.1002/gch2.1007

**Published:** 2015-10-23

**Authors:** Karen A. Grépin

**Affiliations:** ^1^ Robert F. Wagner Graduate School of Public Service New York University New York NY USA

## Abstract

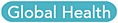

Although it is hard to pin down an exact date of birth, a little more than a decade ago, a new field known widely as global health, was born. Drawing upon the fields of international health and public health, this new community of researchers, practitioners, and advocates focuses on the role of global collective action and policies in improving population health. Spurred by the need to address the most important and pressing global health challenges, the field has been successful in its first few years of life: people are living longer, fewer children are dying before their fifth birthday, and we have made great progress against key infectious diseases.

However, with this growth and with these successes come new challenges, including the need to sustain these gains and to ensure that these improvements are well distributed both across countries and within them. There have also been calls to tackle more global health challenges, including those that are likely to be even more difficult to address, such as the rising burden of non‐communicable diseases. Events from the past few years, most notably global health's abject failure to respond promptly to the Ebola outbreak, suggest that the field may be suffering from growing pains and that we have a lot left to learn. In short, global health is entering into its awkward teenage years.

Given its genetics, in the past when the field has faced a new challenge, leaders tend to search for a technical solution – an “intervention” that is known to be “effective” and can easily be “scaled up”. Many scholarly articles in the top global health journals have made these “calls to action”. But these calls take for granted that all actors care as much about improving population health as those in the field do. The process of generating political priority, adopting new policies, and ensuring that policies have their intended effects requires an understanding of a complex political economy and a methodological toolbox that global health's heritage has ill equipped it to deal with. To continue to grow, global health needs to learn from a more diverse set of voices, and this new journal has an important role to play in this process. I think there are a number of important areas where this journal could help to contribute to ongoing and outstanding debates in the field.

## Who's Afraid of the Big Bad Data?

While almost everyone would agree that good data are a prerequisite to good research, it is a global public good; and like most public goods, there has been too little investment into the production of such goods for global health. At its birth, the situation was even direr and it was nearly impossible to come up with good estimates of what ailed and killed people globally; but today, we have a much better sense of the global burden of disease and the distribution of underlying factors that contribute to this burden globally. However, most of the advancement in our knowledge has come from improvements in our abilities to impute missing data, and there has been much less investment in new sources of data to improve these estimates. In many countries today, there is no reliable source of data on health or health‐seeking behavior; and for many of the other developing countries, we rely upon infrequent (although high quality) household surveys for most of our information. There is nearly no good data on adult illness, and data to track health expenditures, a relatively core concept in health policy, are particularly bad.

In wealthier countries, most policy evaluations are performed using administrative databases, yet few such databases exist or have been shown to be useful in developing countries. New technologies have improved our ability to source health data and have lowered the costs of collecting data. While the rest of the world is moving toward “big data”, global health seems to be stuck in a world of “bad data”. Part of the challenge has been our reluctance to experiment with (and publish) these new data, due to what we see as some of their disadvantages (e.g., a lack of representativeness). However, their advantages may overcompensate for these disadvantages (e.g., timeliness). What can we learn from these new data sources and how can we work together collectively to make data in global health bigger – and better?

## Thinking Beyond Aid Open our Eyes to New Ideas

Given that much of the new programming in global heath over the past decade has been supported by an influx of donor money, it is not surprising that there has been much focus in the field on donors, but this focus has also led to a bias in the types of studies that have been published in global health. Global health is about a lot more than the flow of money from wealthy countries to poorer countries. Rather, and the true value of this epistemic community will come when knowledge generated in more diverse locations by researchers with more diverse backgrounds, and is shared to more diverse audiences. To do this, we need to stop assuming that “we know what works” and rather open our eyes to understand new approaches and to study these experiences systematically.

Improving global health is a complex global challenge and while we have accomplished a lot in our first phase of life, we still have a lot to learn. How can a journal like this help with this process? First, by providing a format and forum for more applied disciplinary as well multi‐disciplinary and inter‐disciplinary scholarship from across the social sciences, we hope that this journal will become the home for important contributions that might not fit well into other leading global health journals. Second, by providing a platform in which other actors involved in addressing other global challenges also contribute, we hope that global health can learn lessons from other sectors and fields. We need to invest more into research that will help us to expand our horizons and learn new approaches. I hope that this journal will help global health along with its intellectual growth in the years ahead.

